# Morpho-physiological and yield traits for selection of drought tolerant *Urochloa* grass ecotypes

**DOI:** 10.1093/aobpla/plae034

**Published:** 2024-06-06

**Authors:** Celestine Anyango Ochola, Mathew Pierro Ngugi, Evans N Nyaboga, Donald M G Njarui

**Affiliations:** Department of Biochemistry, Microbiology and Biotechnology, Kenyatta University, P.O Box 43844-00100, Nairobi, Kenya; Department of Biochemistry, Microbiology and Biotechnology, Kenyatta University, P.O Box 43844-00100, Nairobi, Kenya; Department of Biochemistry, University of Nairobi, P.O Box 30197-00100, Nairobi, Kenya; Kenya Agricultural and Livestock Research Organization (KALRO)-Kabete, P.O Box 14733-00800, Nairobi, Kenya

**Keywords:** Ecotypes, forage, principal component analysis, *Urochloa*, water stress

## Abstract

Drought has become more recurrent and causes a substantial decline in forage yields leading to strain on feed resources for livestock production. This has intensified the search for drought-tolerant forages to promote sustainable livestock production. The objective of this study was to identify drought-tolerant *Urochloa* grasses and to discern their morpho-physiological and yield traits to water stress as well as the relationship between these traits and indices of drought resistance. The results showed that the ecotypes, water regimes and their interaction significantly influenced all the studied morpho-physiological and yield traits. There was a significant decrease in plant height, number of leaves and tillers, dry matter yield, relative water content, photosystem II and efficiency of photosystem II with an increase in non-photochemical quenching. The principal component analysis revealed that the performance of *Urochloa* grass ecotypes was different under water sufficient (WS) and water deficit conditions. Drought tolerance indicators (mean productivity, geometric mean productivity, tolerance index and stress tolerance index) were most effective in identifying *Urochloa* ecotypes with high biomass production under both water deficient and WS conditions. Ecotypes K17, K7, Kisii, Busia and Kakamega were the most drought tolerant, Basilisk, K6, K10, K19 and Toledo were moderately tolerant whereas, CIAT6385, CIAT16449, K13, K5 and K9 were drought sensitive. The five drought-tolerant *Urochloa* ecotypes should be tested for sustainable biomass production under field conditions and used in breeding programmes to develop high-yielding drought-tolerant varieties.

## Teaser

Drought has become frequent due to the changing climatic conditions. Animal feed shortages are rampant during periods of drought. Our study found five drought-tolerant *Urochloa* grass ecotypes that need to be further investigated in the field for sustainable forage grass production under drought-stress conditions. In addition, we identified traits in these grasses that can be utilized in breeding programs to develop high-yielding and drought-tolerant varieties. These forage grasses provide a quality feed option for increased livestock productivity during dry seasons.

## Introduction

The genus *Urochloa* (syn. Brachiaria) has about 135 species that are members of the Poaceae family, Paniceae tribe and Melinidinae subtribe (Ferreira *et al.* 2021). *Urochloa brizantha*, *U. humidicola*, *U. decumbens* and *U. ruziziensis* are the most widely cultivated species in the tropics and sub-tropics because of their economic and agronomic value. *Urochloa* grass grows effectively on infertile acid soils, traps carbon in the soil, fixes nitrogen and minimizes greenhouse gas emissions and ground water pollution by reducing nitrate leaching from agricultural fields (Nandakumar *et al.* 2019; Njarui *et al.* 2020; Van Thanh Ho *et al.* 2020), and therefore, regarded as a climate-smart feed. It also generates a large amount of palatable and nutritious biomass for livestock (Mutimura and Ghimire 2021). *Urochloa* grass has been shown to improve livestock feed availability and thus enhance food and nutrition security (Njarui *et al.* 2020).

The demand for livestock products is high with the world’s meat consumption projected to increase by 8 % and 21 % in developed and developing countries, respectively, by 2027 (OECD 2018). In Kenya, livestock contributes 12 % to the national gross domestic product, and provides food security, income, manure and social-cultural function (GoK 2019; Njarui *et al.* 2020). Livestock production contributes to the realization of United Nations sustainable development goals 1 and 2 including no hunger, provision of food and nutritional security (Schneider and Tarawali 2021). The availability of quality and quantity forage resource is vital for sustainable livestock production (Negawo *et al.* 2017). However, drought reduces forage biomass yield resulting in widespread feed shortage and affect livestock productivity (Djikeng *et al.* 2014; Martin *et al.* 2016; Staver *et al.* 2019). Therefore, the selection of forages that produce high biomass yield under WD is paramount in the face of changing climates (Zuffo *et al.* 2022).

Climate change, especially drought stress, affects the efficiency and productivity of agriculture and subsequently exacerbates food insecurity globally (Dinar *et al.* 2019; Affoh *et al.* 2022; Rohde 2023). Water stress due to drought conditions results in a significant reduction in leaf expansion rate and photosynthesis rate, which inhibits plant development and reduces the biomass production of forage grasses including *Urochloa* grass (Cheruiyot *et al.* 2018; Thaiana *et al.* 2020; Zuffo *et al.* 2022). Therefore, urgent screening of *Urochloa* grass ecotypes under water stress is of fundamental importance in order to identify drought-tolerant varieties that can adapt to future drought conditions. This is critical, especially in arid and semi-arid regions of Kenya, where drought frequency is high. To achieve this, there is a need to comprehend the mechanisms of water stress tolerance in grasses. The identification of *Urochloa* ecotypes with tolerance to drought stress is the first step towards the development of drought-tolerant cultivars. Forage plants exposed to WD during their growth and development must adapt in order to cope with the environmental conditions. As a result, plants under water stress respond through morpho-physiology modifications to withstand the stress and avoid cell damage (Mastalerczuk and Borawska-Jarmułowicz 2021).

Morpho-physiological and yield responses to drought for each forage grass species or cultivar are dependent on the plant genetic characteristics and phenotype (Staniak and Kocoń 2015; Zahid *et al.* 2021; Zhang, *et al.* 2022). The photosynthetic parameters affected by drought stress include efficiency for photosystem II (*F*_*v*_/*F*_*m*_), relative chlorophyll content (SPAD), photosystem II photochemistry (Phi2) and non-photochemical quenching (PhiNPQ, Kuhlgert *et al.* 2016; De Souza *et al.* 2021). WD significantly reduces photosynthesis that hinders plant growth and lowers the biomass yield of grasses (Staniak 2016; Fariaszewska *et al.* 2020). The associations between dry matter yield (DMY) and the morpho-physiological variables, however, are not always direct and clear. The adaptation of plants to drought is influenced by their ability to maintain normal chlorophyll fluorescence or photosynthetic features under drought stress (Chen *et al.* 2016). The effectiveness of breeding programmes in water-stressed areas can be increased by understanding the relationships between yield, morpho-physiological traits and selection indices within a plant ecotype. The aim of this study was to identify potential drought-tolerant *Urochloa* spp. germplasm and to discern their morpho-physiological and yield traits to water stress as well as the relationship between these traits and indices of drought resistance.

## Materials and Methods

### Experimental site and plant materials

The drought stress experiment was conducted in a screen house at Kenya Agricultural and Livestock Research Organization (KALRO), Katumani station (37° 28ʹ E, 1° 58ʹ S and 1600 m above sea level) in between April and August 2021. A total of 35 *Urochloa* ecotypes grass obtained from KALRO were used in this study [**see Supporting Information—** Table S1]. The ecotypes were originally obtained from different geographical locations in Kenya and preserved at KALRO—Katumani. The 33 ecotypes selected for use in this study were collected from semi-arid regions that are characterized by frequent droughts. Three ecotypes namely *Urochloa decumbens* cv. Basilisk, *Urochloa brizantha* cvs Toledo and Piata were included as controls due to the fact that they have been reported to produce comparatively high fodder yield under conditions of drought stress (Njarui *et al.* 2016; Cheruiyot *et al.* 2018).

### Experimental design, water treatments and growing conditions

The design of the experiments was a randomized complete block in split-plot arrangement with five replications. Two water regime used in this study: water sufficient (WS) ~45 % volumetric water content (VWC) and water deficit (WD) ~11 % VWC. The two WR were selected due to the fact that they have been reported to be efficient in the selection of drought-tolerant plants including *Urochloa* grass ecotypes (Cheruiyot *et al.* 2018; Marchin *et al.* 2020). We also performed preliminary trials at ~45 % and ~11 % VMC to test the efficacy of the two WR, which proved to be efficient in the classification of ecotypes into different categories of drought tolerance.

Because of limited seeds, each *Urochloa* grass ecotype was propagated using rooted tillers. The rooted tillers of approximately the same size and age from each ecotype were selected, and one tiller was transplanted per plastic pot (11.5 cm × 15 cm) with eight holes at the bottom for drainage. The pots were labelled by ecotype name, filled with 1 kg sterile forest soil (N 0.17, C 1.0, P 10, K 0.76, Mg 3.0, Ca 2.0, Mn 0.32 Cu 4.32, Fe 10.7, Zn 0.67 and Na 0.43) and sand mixed in the ratio of 3:2 to improve drainage. The plants were then watered daily to 100 % of the maximal VWC. After 4 weeks, a standardization cut was made at 5 cm above the soil. The plants were then maintained at 100 % field water capacity (FC) (~45 % VWC) for 21 days and developed between 3 and 4 leaves prior to water stress treatments. The plants were grown in the greenhouse under natural light. The temperature ranged from 17.2 °C to 18.5 °C, from 35.3 °C to 40.4°C, and from 29.6 °C to 30.7°C, while humidity ranged from 60% to 75 %, 28.3–29.7 % and 31.5–33.2 % at 8.00 a.m., 12 p.m. and 5 p.m., respectively.

Plants under WS condition were watered daily to 45 % VWC. However, watering was stopped for the experimental plants under WD to enable progressive drying of the soil to achieve ~11 % VWC as described in Marchin *et al*. (2020). The soil VWC of each plastic pot in the WD treatments before and during the experimental period was measured daily using a soil moisture sensor (Procheck Decagon Device, Inc PC 157C with GS3). Pots exceeding the upper limit of the targeted drought intensity per day (>11 % VWC) were allowed time to drain to achieve the targeted intensity. If soil VWC surpassed the lower limit of the targeted intensity, water was added to the soil surface of the pot to maintain the targeted soil VWC. The specific soil VWC (~11 % VWC) was then maintained for a period of 28 days. Poorter *et al*. (2012) recommended drought treatment period of ~28 days as adequate to ensure total plant dry weight to pot volume less than 2 kg m^3^.

### Measurement of morphological traits

After 28 days of water stress treatment, morphological traits were measured. In both the WD and WS conditions, three plants of each ecotype were randomly selected from each pot. The morphological data collected included the number of tillers (NT) per pot and plant height (PH) of tallest tiller in each pot measured above the soil using a wooden ruler. The number of leaves (NL) in the primary tiller was counted. The plants were uprooted, roots cleaned in tap water and root length (RL) measured.

### Measurement of physiological traits

In order to assess the impact of the soil WD on plant physiological function, one plant per replication was selected and used for analysis. The youngest fully expanded leaf was used to determine the relative water content (RWC) as described by Chen *et al*. (2016). Fresh weight (FWT) was immediately determined by weighing the youngest fully expanded leaf. Leaf segments were then rehydrated in distilled water for 6 h in a closed container in the dark to determine turgid weight (TW) while the DMY was measured after leaf segments is dried at 65 °C in an oven for 48 h.


$$\text{Relative water content}=\left(\frac{\text{FWT}-\text{DMY}}{\text{TW}-\text{DMY}}\right)\times 100\%$$

Chlorophyll florescence-based photosynthetic parameters including photosystem II photochemistry (Phi2), non-photochemical quenching (PhiNPQ), relative chlorophyll content (SPAD) and quantum yield efficiency for photosystem II (*F*_*v*_/*F*_*m*_) were measured using photosynq multispeQ instrument (v1.0) linked to the PhotosynQ platform (http://www.photosynq.com/technology) (Kuhlgert *et al.* 2016; Putranto 2018). In each water treatment, one plant per ecotype in each replication was randomly sampled and chlorophyll florescence-based photosynthetic parameters determined from the centre of the last fully expanded leaf without altering the leaf angle. The multispeQ protocol used during this study was Photosynthesis RIDES 2.0.

### Biomass yield and calculation of drought tolerance indices

All the plant leaves and stems of each ecotype per replication were cut and weighed using analytical balance to determine the FWT. DMY was determined after drying the shoot samples in the oven at 65 °C for 72 h. Drought tolerance indices (DTI) based on biomass yield under the two WR (WD and WS) were calculated as follows:

$Mean\;\;\;productivity\;\;\;(MP)=\frac{Ys+Yp}{2}$ (Naghavi *et al.* 2013)

Geometric   mean   productivity   (GMP)=√(Ys×Yp) (Majidi *et al.* 2016)

Tolerance   index   (TOL)=   Yp−Ys (Menezes *et al.* 2014)

$Yield\;\;\;stability\;\;\;index\;\;\;(YSI)=\frac{Ys}{Yp}\;\;\;$ (Naghavi *et al.* 2013)

$Yield\;\;\;index\;\;\;(YI)=\frac{Ys}{\bar{Y}s}$ (Naghavi *et al.* 2013)

$Stress\;\;\;susceptibility\;\;\;index\;\;\;(SSI)=\frac{1-Ys/Yp}{1-\bar{Y}s/\bar{Y}p}$ (Fischer and Maurer 1978)

$Stress\;\;\;Tolerance\;\;\;Index\;\;\;(STI)=\frac{Ys\times Yp}{\bar{Y}p^{2}}$ (Zuffo *et al.* 2022)

where Ys is the ecotype yield under WD; Yp is the ecotype yield under water sufficient; Ȳs is the mean yield of all ecotypes under WD; and Ȳp is the mean yield of all ecotypes under WS condition.

### Statistical analysis

The General Linear Model procedure in R (version 4.1.2) (R Core Team 2021) was used for the analysis of variance (ANOVA) for the morpho-physiological and yield traits. Mean separation was done using the least significant difference (LSD) (*P* ≤ 0.05) to test the genotypic difference, drought stress effect and to compare the phenotypic value of the genotype for specific trait and different WR. FactoMineR and Factoextra were used to create principal component analysis (PCA) biplots using all the measured and calculated variables (Kassambara and Mundt 2017; Chaouachi *et al.* 2023). Agglomerative hierarchical clustering was done using the Euclidean distance algorithm. Ranking using PCA was applied to assess drought tolerance level of each *Urochloa* ecotype. Ranking value was calculated using a formula given by Aghaie *et al*. (2018) and Ajtahed *et al*. (2021) as follows:


$$\begin{array}{lllll} Ranking\;value= & \;[(contribution\;\;\;of\;\;\;PC1(\;\%\;)\times PC1)\; & +(contribution\;\;\;of\;\;\;PC2(\;\%\;)\times PC2)\; & +(contribution\;\;\;of\;\;\;PC3(\;\%\;)\times PC3)\; & +(contribution\;\;\;of\;\;\;PC4(\;\%\;)\times PC4)]. \end{array}$$

The contributions of the four primary components PC1, PC2, PC3 and PC4 were determined by PCA analysis and are represented as % in this formula. The PC1, PC2, PC3 and PC4 are the PCA loading of morpho-physiological and yield traits for 35 *Urochloa* ecotypes subjected to WD conditions. A numerical rank was calculated from the mean ranking values under WD conditions.

## Results

### Effects of drought stress on morpho-physiological and yield traits

The ANOVA revealed significant (*P* < 0.001) variation among Urochloa grass ecotypes for all the studied morpho-physiological and yield traits [see Supporting Information—Table S2]. The water regimes (WR) were the largest contributor to the variation observed across all the traits (see Supporting Information—Table S2; *P* < 0.001). Moreover, the interaction between Eco × WR was significant for all the traits [see Supporting Information—Table S2].

Morpho-physiological traits and DM yield were significantly (*P* < 0.05) reduced in plants exposed to WD, while non-photochemical quenching (PhiNPQ) significantly increased under WD (Tables 1 and 2 and Supporting Information—Table S3). The percentage reductions varied in studied traits, with RWC, relative chlorophyll content (SPAD) and fresh weight (FWT) recording significantly high reductions of 85.9 %, 85.3 % and 84 %, respectively, under WD conditions. The NT per plant, followed by the NL, and RL, was the least affected by WD conditions (Table 1).

**Table 1.** Mean PH, NT, number of leaves and root length *Urochloa* grass ecotypes under WS and WD. CV, coefficient of variation; LSD, least significance difference. Values expressed as Mean ± SEM (*n* = 3)

Traits	Plant height (cm)		Number of tillers		Number of leaves		Root length (cm)	
Ecotypes	WS	WD	WS	WD	WS	WD	WS	WD
CIAT16449	8.66 ± 0.21	5.6 ± 0.25	2.6 ± 0.60	1.4 ± 0.25	4.4 ± 0.40	2.8 ± 0.37	54.8 ± 0.20	44.4 ± 2.25
CIAT16514	25.04 ± 0.16	10.4 ± 0.25	4.6 ± 0.25	6.20.37	4.2 ± 0.37	3.2 ± 0.80	90.8 ± 0.20	42.4 ± 5.06
CIAT 6384	11.00 ± 0.32	8.4 ± 0.25	3.0 ± 0.45	2.8 ± 0.37	5.4 ± 0.51	2.8 ± 0.37	85.0 ± 1.67	57.4 ± 5.99
CIAT 6385	8.60 ± 0.25	7.8 ± 0.26	1.2 ± 0.20	2.0 ± 0.00	3.2 ± 0.20	3.0 ± 0.45	111.2 ± 0.58	55.2 ± 13.9
CIAT6399	17.46 ± 0.16	4.6 ± 0.25	7.2 ± 0.20	5.0 ± 0.32	5.4 ± 0.40	2.6 ± 0.25	90.6 ± 0.25	48.2 ± 4.74
CIAT6426	15.7 ± 0.26	6.5 ± 0.22	2.2 ± 0.49	1.8 ± 0.37	4.4 ± 0.24	3.2 ± 0.20	76.6 ± 0.93	49.2 ± 6.06
CIAT6684	10.82 ± 0.23	9.0 ± 0.27	3.0 ± 0.00	1.2 ± 0.20	4.2 ± 0.74	3.2 ± 0.20	51.0 ± 0.45	47.8 ± 3.18
cv. Basilisk	18.5 ± 0.22	7.8 ± 0.30	11.8 ± 0.20	4.8 ± 0.20	5.8 ± 0.37	2.8 ± 0.20	79.6 ± 4.89	57.0 ± 4.09
Busia	13.9 ± 0.19	7.9 ± 0.18	7.6 ± 0.25	6.6 ± 0.25	6.2 ± 0.49	4.0 ± 0.00	55.4 ± 0.25	54.8 ± 5.42
K1	24.1 ± 0.40	11.5 ± 0.32	4.6 ± 0.25	3.4 ± 0.40	5.8 ± 0.37	4.6 ± 0.40	80.0 ± 0.32	43.4 ± 4.28
K2	16.60 ± 0.37	12.2 ± 0.52	6.6 ± 0.25	6.8 ± 0.37	4.4 ± 0.25	2.8 ± 0.37	77.0 ± 0.45	43.6 ± 9.41
K3	12.8 ± 0.12	11.0 ± 0.61	3.2 ± 0.20	2.0 ± 0.32	4.0 ± 0.32	4.4 ± 0.40	74.4 ± 0.25	53.2 ± 7.08
K4	15.46 ± 0.17	11.6 ± 0.29	3.4 ± 0.25	2.4 ± 0.25	4.2 ± 0.37	3.6 ± 0.25	82.0 ± 0.00	50.2 ± 6.64
K5	12.56 ± 0.25	5.2 ± 0.20	2.8 ± 0.20	1.4 ± 0.25	4.0 ± 0.32	2.4 ± 0.40	50.6 ± 0.40	55.0 ± 1.48
K6	18.00 ± 0.27	6.4 ± 0.25	4.2 ± 0.86	5.0 ± 0.32	5.2 ± 0.92	3.0 ± 0.45	72.6 ± 4.37	53.0 ± 4.72
K7	29.90 ± 0.33	12.5 ± 0.22	6.6 ± 0.25	3.8 ± 0.20	5.2 ± 0.58	4.2 ± 0.20	87.2 ± 0.37	62.6 ± 3.47
K8	12.54 ± 0.23	6.6 ± 0.25	5.0 ± 0.71	4.2 ± 0.20	6.0 ± 0.55	2.8 ± 0.20	97.0 ± 0.55	51.0 ± 2.30
K9	11.54 ± 0.25	6.5 ± 0.32	2.8 ± 0.20	2.2 ± 0.37	5.0 ± 0.55	2.8 ± 0.49	121.8 ± 6.21	53.0 ± 11.6
K10	22.0 ± 0.55	7.6 ± 0.25	5.6 ± 0.25	4.4 ± 0.25	5.8 ± 0.37	3.6 ± 0.40	68.2 ± 0.49	69.2 ± 4.22
K12	16.3 ± 0.37	7.7 ± 9.20	3.8 ± 0.20	1.4 ± 0.25	4.2 ± 0.20	3.6 ± 0.25	87.4 ± 0.40	42.0 ± 3.89
K13	7.08 ± 0.11	5.2 ± 0.37	2.2 ± 0.20	2.2 ± 0.20	4.6 ± 0.25	2.8 ± 0.37	53.0 ± 6.20	55.4 ± 8.62
K15	7.64 ± 0.09	4.6 ± 0.19	7.2 ± 0.20	9.0 ± 0.00	4.8 ± 0.58	4.6 ± 0.40	55.6 ± 0.25	50.6 ± 5.62
K16	11.62 ± 0.18	8.8 ± 0.12	3.0 ± 0.71	2.0 ± 0.45	4.8 ± 0.66	3.2 ± 0.49	84.8 ± 1.24	49.0 ± 7.63
K17	21.20 ± 0.37	15.8 ± 0.26	5.8 ± 0.20	4.2 ± 0.37	4.6 ± 0.25	4.0 ± 0.32	71.8 ± 3.81	62.0 ± 6.31
K18	14.72 ± 0.19	5.7 ± 0.30	4.0 ± 0.00	1.6 ± 0.45	5.0 ± 0.32	3.4 ± 025	68.6 ± 0.25	63.2 ± 10.9
K19	17.50 ± 0.22	11.5 ± 0.32	8.4 ± 0.25	3.2 ± 0.37	5.6 ± 0.25	3.6 ± 0.25	75.2 ± 0.37	52.6 ± 6.53
K20	7.80 ± 0.20	6.4 ± 0.25	2.4 ± 0.25	1.8 ± 0.37	5.0 ± 0.32	3.8 ± 0.37	62.2 ± 0.20	53.6 ± 8.39
K21	19.60 ± 0.25	11.6 ± 0.25	3.4 ± 0.25	2.8 ± 0.20	4.0 ± 0.32	3.2 ± 0.20	74.4 ± 0.25	45.8 ± 4.45
K22	24.50 ± 0.22	11.6 ± 0.25	5.0 ± 0.95	5.2 ± 0.20	5.6 ± 0.25	3.4 ± 0.25	58.4 ± 0.25	47.8 ± 2.60
K23	8.12 ± 0.20	5.5 ± 0.32	10.6 ± 0.25	9.0 ± 0.32	5.6 ± 0.40	4.2 ± 0.20	61.4 ± 1.66	63.6 ± 7.35
Kakamega	11.20 ± 0.52	8.6 ± 0.19	5.6 ± 0.25	4.8 ± 0.37	3.6 ± 0.25	3.0 ± 0.00	85.4 ± 1.86	47.6 ± 2.50
Kisii	11.90 ± 0.19	6.8 ± 0.26	11.6 ± 0.40	9.0 ± 0.32	5.2 ± 0.37	3.6 ± 0.25	69.0 ± 0.20	42.2 ± 1.24
Lanet	20.00 ± 0.35	13.0 ± 0.16	6.6 ± 0.25	3.8 ± 0.37	5.6 ± 0.25	4.2 ± 0.20	95.2 ± 0.74	44.6 ± 4.52
cv. Piata	6.40 ± 0.29	4.6 ± 0.25	4.2 ± 0.20	3.0 ± 0.32	4.0 ± 0.95	3.6 ± 0.25	69.8 ± 0.20	36.0 ± 1.26
cv. Toledo	11.0 ± 0.16	6.4 ± 0.24	5.8 ± 0.97	4.2 ± 0.37	3.6 ± 0.40	2.8 ± 0.20	67.4 ± 2.94	63.4 ± 3.41
Mean	14.9 ± 0.44	8.37 ± 0.22	5.07 ± 0.21	3.85 ± 0.17	4.82 ± 0.09	3.39 ± 0.07	75.53 ± 1.27	51.72 ± 1.09
% CV	38.0	34.7	53.70	59.3	24.5	26.6	22.3	27.8
LSD_0.05_	0.76	0.79	1.13	0.85	1.25	0.95	5.94	17.31

**Table 2.** Mean physiological traits for *Urochloa* grass ecotypes under WS and WD conditions.

Traits	Relative water content (RWC)		Photosystem II (Phi2)		Non-photochemical quenching (PhiNPQ)		Relative chlorophyll content (SPAD)		Efficiency of photosystem II (*F*_v_/*F*_m_)	
Ecotypes	WS	WD	WS	WD	WS	WD	WS	WD	WS	WD
CIAT16449	81.66 ± 1.60	24.59 ± 1.14	0.45 ± 0.01	0.21 ± 0.03	0.57 ± 0.06	0.74 ± 0.04	43.62 ± 3.02	2.14 ± 0.42	0.43 ± 0.04	0.26 ± 0.03
CIAT16514	73.66 ± 0.44	12.68 ± 0.65	0.33 ± 0.10	0.19 ± 0.04	0.56 ± 0.11	0.75 ± 0.05	33.42 ± 1.90	4.72 ± 0.31	0.44 ± 0.08	0.27 ± 0.05
CIAT6384	68.30 ± 3.41	21.50 ± 13.8	0.56 ± 0.06	0.20 ± 0.03	0.69 ± 0.05	0.75 ± 0.03	36.59 ± 0.81	3.46 ± 0.93	0.50 ± 0.06	0.24 ± 0.03
CIAT6385	75.14 ± 1.08	13.31 ± 1.42	0.42 ± 0.08	0.24 ± 0.04	0.59 ± 0.12	0.69 ± 0.05	26.98 ± 1.22	2.97 ± 0.66	0.48 ± 0.09	0.33 ± 0.04
CIAT6399	89.96 ± 1.69	6.77 ± 0.94	0.37 ± 0.05	0.23 ± 0.04	0.63 ± 0.03	0.71 ± 0.04	37.18 ± 1.03	2.74 ± 0.35	0.45 ± 0.04	0.27 ± 0.03
CIAT6426	70.23 ± 1.12	16.46 ± 0.44	0.45 ± 0.02	0.14 ± 0.02	0.39 ± 0.10	0.82 ± 0.02	37.46 ± 1.32	11.16 ± 1.12	0.46 ± 0.05	0.18 ± 0.02
CIAT6684	72.40 ± 0.66	17.72 ± 0.25	0.17 ± 0.03	0.15 ± 0.02	0.39 ± 0.09	0.81 ± 0.03	43.75 ± 0.86	3.38 ± 0.71	0.32 ± 0.04	0.20 ± 0.03
cv. Basilisk	80.74 ± 0.55	18.10 ± 0.64	0.54 ± 0.02	0.20 ± 0.04	0.70 ± 0.04	0.75 ± 0.03	35.13 ± 0.93	3.13 ± 0.67	0.45 ± 0.05	0.24 ± 0.04
Busia	86.63 ± 0.59	7.09 ± 0.61	0.26 ± 0.01	0.14 ± 0.00	0.64 ± 0.02	0.82 ± 0.01	42.75 ± 0.22	4.12 ± 0.56	0.47 ± 0.04	0.18 ± 0.01
K1	77.73 ± 0.43	9.96 ± 1.38	0.34 ± 0.01	0.12 ± 0.03	0.70 ± 0.04	0.84 ± 0.03	38.41 ± 0.16	13.70 ± 1.02	0.39 ± 0.03	0.17 ± 0.03
K2	93.62 ± 0.89	12.28 ± 0.22	0.53 ± 0.04	0.21 ± 0.01	0.45 ± 0.04	0.72 ± 0.01	38.76 ± 1.66	13.11 ± 0.34	0.46 ± 0.05	0.28 ± 0.01
K3	66.77 ± 0.43	5.08 ± 0.84	0.26 ± 0.02	0.14 ± 0.01	0.56 ± 0.06	0.82 ± 0.02	36.31 ± 0.59	12.08 ± 1.25	0.43 ± 0.01	0.18 ± 0.02
K4	75.62 ± 0.39	16.61 ± 61	0.32 ± 0.05	0.12 ± 0.01	0.65 ± 0.04	0.84 ± 0.02	40.54 ± 0.84	6.57 ± 1.86	0.41 ± 0.02	0.19 ± 0.02
K5	64.71 ± 0.43	2.46 ± 0.33	0.30 ± 0.05	0.21 ± 0.02	0.68 ± 0.03	0.74 ± 0.02	34.59 ± 0.19	2.48 ± 0.53	0.38 ± 0.06	0.20 ± 0.02
K6	63.79 ± 0.13	9.65 ± 1.04	0.56 ± 0.06	0.22 ± 0.04	0.71 ± 0.06	0.72 ± 0.03	30.70 ± 1.27	6.66 ± 0.23	0.44 ± 0.09	0.27 ± 0.02
K7	74.35 ± 0.82	9.61 ± 2.21	0.47 ± 0.01	0.11 ± 0.01	0.67 ± 0.06	0.86 ± 0.02	34.51 ± 1.70	12.12 ± 1.00	0.37 ± 0.02	0.15 ± 0.02
K8	76.58 ± 0.54	10.88 ± 1.81	0.40 ± 0.03	0.21 ± 0.01	0.67 ± 0.03	0.73 ± 0.01	32.43 ± 0.88	9.31 ± 0.29	0.47 ± 0.02	0.26 ± 0.01
K9	85.43 ± 0.90	11.56 ± 0.39	0.49 ± 0.04	0.32 ± 0.04	0.51 ± 0.09	0.57 ± 0.05	36.23 ± 0.48	1.92 ± 0.42	0.51 ± 0.03	0.44 ± 0.03
K10	74.18 ± 0.67	8.97 ± 2.31	0.68 ± 0.01	0.19 ± 0.02	0.64 ± 0.04	0.76 ± 0.03	36.45 ± 1.14	2.06 ± 0.37	0.55 ± 0.08	0.25 ± 0.03
K12	62.32 ± 1.05	8.99 ± 1.22	0.45 ± 0.01	0.17 ± 0.02	0.58 ± 0.02	0.78 ± 0.30	33.01 ± 1.50	5.88 ± 0.30	0.42 ± 0.04	0.22 ± 0.03
K13	64.27 ± 2.47	10.0 ± 0.62	0.23 ± 0.01	0.16 ± 0.01	0.72 ± 0.03	0.80 ± 0.02	33.62 ± 0.97	3.13 ± 0.53	0.30 ± 0.04	0.20 ± 0.01
K15	61.32 ± 0.57	5.75 ± 0.65	0.25 ± 0.03	0.16 ± 0.02	0.59 ± 0.06	0.79 ± 0.03	48.48 ± 0.20	1.44 ± 0.16	0.41 ± 0.03	0.21 ± 0.02
K16	74.41 ± 3.36	8.19 ± 0.50	0.24 ± 0.01	0.20 ± 0.05	0.59 ± 0.06	0.74 ± 0.07	34.60 ± 1.17	4.41 ± 1.82	0.36 ± 0.02	0.26 ± 0.05
K17	55.68 ± 1.06	6.74 ± 1.09	0.39 ± 0.02	0.14 ± 0.03	0.66 ± 0.01	0.82 ± 0.04	32.33 ± 0.52	8.33 ± 0.88	0.42 ± 004	0.18 ± 0.03
K18	55.68 ± 1.42	3.28 ± 0.81	0.16 ± 0.01	0.15 ± 0.02	0.75 ± 0.04	0.81 ± 0.03	42.10 ± 0.53	3.11 ± 0.37	0.37 ± 0.04	0.19 ± 0.03
K19	64.97 ± 0.91	5.51 ± 0.84	0.23 ± 0.01	0.16 ± 0.03	0.61 ± 0.06	0.79 ± 0.03	36.61 ± 0.52	2.85 ± 0.70	0.28 ± 0.04	0.21 ± 0.03
K20	68.46 ± 1.15	8.94 ± 0.54	0.37 ± 0.05	0.15 ± 0.01	0.41 ± 0.06	0.81 ± 0.01	33.92 ± 0.81	2.94 ± 0.46	0.37 ± 0.06	0.19 ± 0.01
K21	83.73 ± 1.33	13.36 ± 0.42	0.36 ± 0.03	0.17 ± 0.04	0.67 ± 0.03	0.78 ± 0.05	37.14 ± 0.65	11.30 ± 0.46	0.42 ± 0.05	0.23 ± 0.05
K22	68.11 ± 1.39	10.74 ± 1.32	0.27 ± 0.02	0.36 ± 0.09	0.46 ± 0.06	0.52 ± 0.12	36.83 ± 2.07	2.45 ± 0.08	0.38 ± 0.03	0.46 ± 0.09
K23	68.05 ± 0.36	4.18 ± 0.85	0.21 ± 0.02	0.18 ± 0.02	0.55 ± 0.02	0.77 ± 0.03	38.12 ± 2.16	1.45 ± 0.11	0.42 ± 0.05	0.24 ± 0.03
Kakamega	76.20 ± 0.32	6.72 ± 0.53	0.31 ± 0.02	0.16 ± 0.02	0.66 ± 0.03	0.79 ± 0.02	42.09 ± 0.74	5.28 ± 0.71	0.40 ± 0.02	0.21 ± 0.01
Kisii	91.56 ± 1.72	10.67 ± 0.38	0.34 ± 0.04	0.07 ± 0.00	0.52 ± 0.03	0.90 ± 0.00	37.89 ± 0.55	8.12 ± 1.98	0.47 ± 0.01	0.12 ± 0.01
Lanet	71.62 ± 0.29	5.61 ± 1.73	0.37 ± 0.04	0.17 ± 0.02	0.64 ± 0.04	0.78 ± 0.03	33.11 ± 1.80	4.90 ± 0.86	0.34 ± 0.09	0.22 ± 0.02
cv. Piata	88.96 ± 0.22	10.69 ± 1.11	0.25 ± 0.02	0.16 ± 0.01	0.63 ± 0.05	0.80 ± 0.02	45.11 ± 0.53	2.36 ± 0.36	0.26 ± 0.09	0.19 ± 0.01
cv. Toledo	92.97 ± 0.70	11.39 ± 0.23	0.19 ± 0.01	0.23 ± 0.02	0.68 ± 0.03	0.70 ± 0.03	39.37 ± 1.46	4.51 ± 0.85	0.40 ± 0.02	0.31 ± 0.02
Mean	74.27 ± 0.79	10.46 ± 0.54	0.36 ± 0.01	0.18 ± 0.01	0.60 ± 0.01	0.77 ± 0.01	37.14 ± 0.38	5.44 ± 0.30	0.41 ± 0.01	0.23 ± 0.00
%CV	14.05	68.05	35.25	44.05	24.06	13.48	13.53	73.89	28.91	39.76
LSD	3.45	7.12	0.104	0.082	0.16	0.10	3.46	2.31	0.14	0.27

Drought stress led to a significant decrease in PH by 43.8 %. The highest decrease was observed for CIAT6399 (73.7 %), K10 (65.5 %) and K6 (64.4 %). Ecotypes K7 and K17 were significantly taller than all the other ecotypes under WS and WD conditions, respectively, (Table 1; *P* ≤ 0.05). The NT was significantly reduced by WD for most of the studied ecotypes, except CIAT16514, CIAT6385, K2, K6, K15 and K22. Ecotypes K12 and K19 recorded significant NT decreases of 63.2 % and 61.9 %, respectively (Table 1; *P* ≤ 0.05). Kisii, K15 and K23 had significantly higher NT than all the other ecotypes while CIAT6684 recorded the lowest NT under WD conditions. WD significantly reduced NL by 29.7 %. Ecotypes K1 and K15 recorded significantly high NL (Table 1). Decreased RL was observed under WD conditions (*P* ≤ 0.05), with CIAT16514, K8, and Lanet recording high percentage reductions of 53.3 %, 56.5 % and 53.2 %, respectively.

The RWC significantly decreased under WD conditions (Table 2; *P* ≤ 0.05). Ecotypes K5 (96.2 %) and K18 (94.1 %) recorded the highest percentage reductions. A significant high RWC was recorded by CIAT16449 under WD conditions, whereas K5 recorded the lowest value (Table 2; *P* ≤ 0.05). In addition, a significant reduction of 50 % was observed in Phi 2 under WD conditions (Table 2). The highest decrease was recorded by Kisii (79.4 %) and K7 (76.6 %), whereas significant high values were observed in K22 and K9. WD significantly increased PhiNPQ by 28.3 % (Table 2; *P* ≤ 0.05). CIAT6426 recorded the highest increase in PhiNPQ by 110.3 % and cv. Toledo the lowest (2.9 %). For Phi2, PhiNPQ, SPAD and *F*_v_/*F*_m_, significant high values were observed in ecotype K22, Kisii, K1 and K22, respectively, under WD conditions (Table 2; *P* ≤ 0.05). The SPAD significantly declined under WD (*P* ≤ 0.05), with K2 recording the highest value. Moreover, a significant decline of 43.9 % was observed in *F*_*v*_/*F*_m_ ratio under water stress. Ecotype K22 and K9 recorded the highest *F*_*v*_/*F*_m_ under WD. The average DMY decreased by 66.8 %, with the Kakamega ecotype recording a significantly higher DM yield under WD than the other ecotypes [see Supporting Table S3].

### Association among morpho-physiological traits and biomass yield

**Figure 1.**
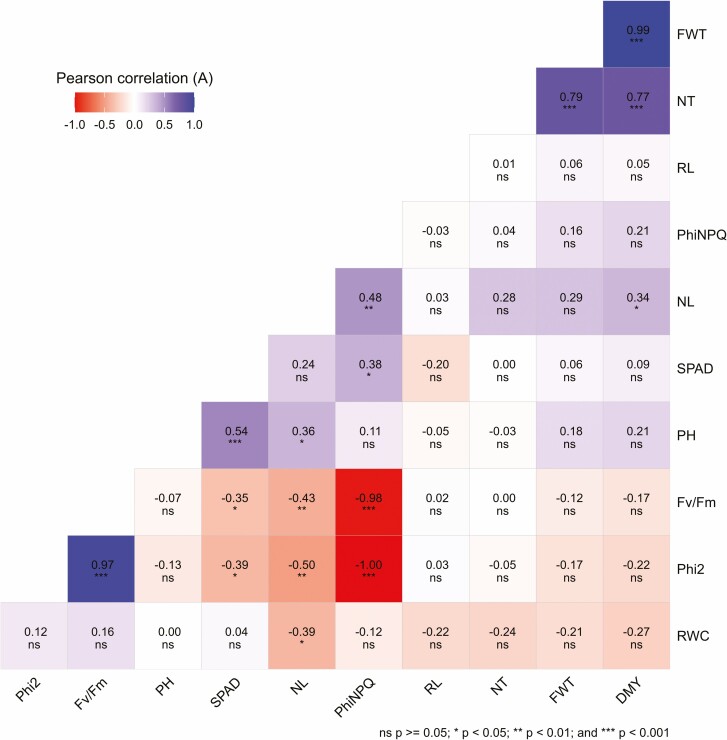

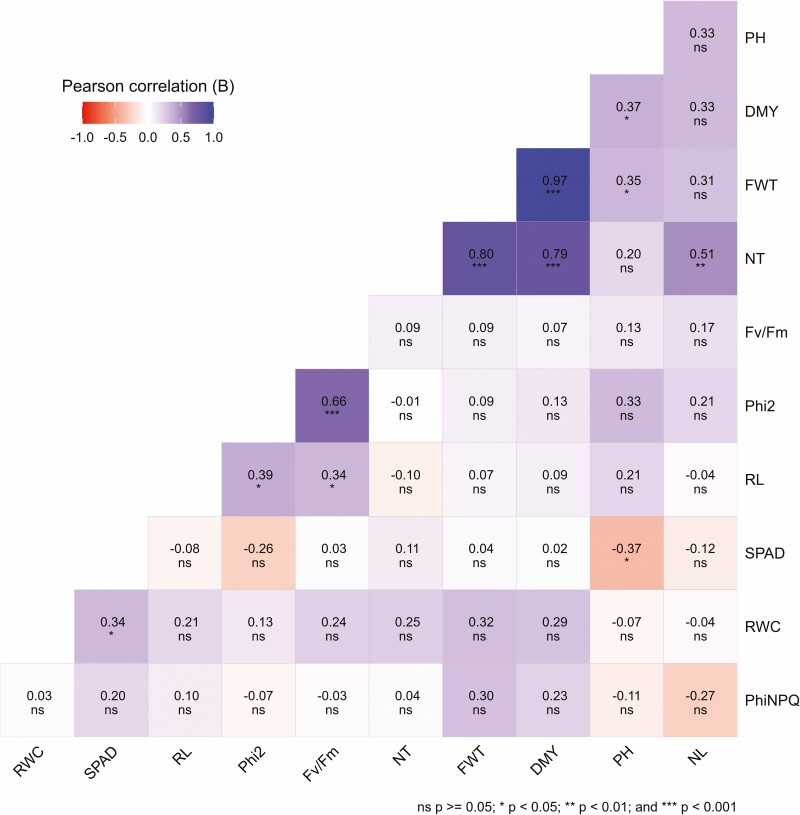
Correlation plot for morpho-physiological and yield traits under WD (A) and WS (B) conditions. NT, number of tillers; PH, plant height; NL, number of leaves; RL, root length; DMY, dry matter yield; FWT, fresh weight; RWC, relative water content; Phi2, photosystem II photochemistry; PhiNPQ, non-photochemical quenching, SPAD, relative chlorophyll content; *F*_*v*_/*F*_*m*_, efficiency of photosystem II.

Under WD conditions, DMY was significantly and positively correlated with NT (*P* < 0.001) and NL (*P* ≤ 0.05; Fig. 1A). Additionally, NL was positively associated with PH and PhiNPQ but negatively correlated with RWC, Phi2 and *F*_*v*_/*F*_m_. Relative chlorophyll content (SPAD) was positively associated with PH and PhiNPQ but negatively correlated with Phi2 and *F*_*v*_/*F*_*m*_. A significant positive correlation was also observed between *F*_v_/*F*_m_ with Phi2 while PhiNPQ was negatively associated with Phi2 and *F*_*v*_/*F*_*m*_ under WD (Fig. 1A).

Under WS, morpho-physiological traits showed significant and positive correlations between PH with DMY, whereas, SPAD was negatively associated with PH (*P* < 0.05; Fig. 1B). Moreover, there was a significant positive association between DMY with NT and NL. RL was significantly and positively correlated with Phi2 and *F*_*v*_/*F*_*m*_ (*P* ≤ 0.05). The DYM and FWT; Phi2 and Fv/Fm; RWC and SPAD were positively correlated under WS conditions (Fig. 1B).

Yield under WS (Yp) was significantly and positively associated with yield under WD (Ys) conditions [see Supporting Information Table S4]. The data showed that MP, GMP, TOL, STI and YI were positively associated with Yp and Ys. SSI was negatively correlated with Ys but not significantly associated with Yp. In contrast, YSI was negatively correlated with all the drought indices tested in this study [see Supporting Information Table S4].

### Principal component analysis biplots for morpho-physiological and biomass yield traits

**Figure 2.**
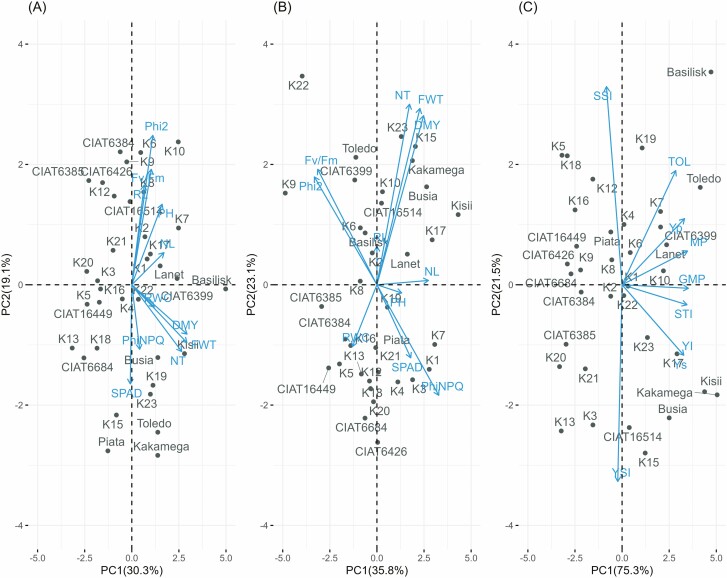
PCA biplot illustrating contribution of all the studied traits under WS (A), WD (B) conditions and DTI and the distribution of the 35 *Urochloa* grass ecotypes (C). NT, number of tillers; PH, plant height; NL, number of leaves; RL, root length; DMY, dry matter yield; RWC, relative water content; Phi2, photosystem II photochemistry; PhiNPQ, non-photochemical quenching; SPAD, relative chlorophyll content; *F*_*v*_/*F*_*m*_, efficiency of photosystem II; MP, mean productivity; GMP, geometric mean productivity; TOL, tolerance index;YSI, yield stability index; YI, yield index; Yp, mean biomass yield under WS; Ys, mean biomass yield under WD, STI, stress tolerance index; SSI, tress susceptible index.

The first two PCA explained cumulative variance of 49.4 % and 58.9 % under WS and WD conditions, respectively (Fig. 2). It is evident that PH, NL, RL, NT, RWC, DMY, Phi2, *F*_*v*_/*F*_*m*_, PhiNPQ and SPAD parameters have a role in the variability of the *Urochloa* germplasm (Fig. 2). Under WS conditions, DMY, NT and PH showed significant positive contributions while PhiNPQ and SPAD contributed negatively in PC1 (Fig. 2A). The PC2 was associated with diversity among *Urochloa* ecotypes due to *F*_*v*_/*F*_m_, Phi2, RL and SPAD. Kisii and Busia cultivars had the highest biomass yield as well as RWC, which are related to the NT per plant. In addition to the RL, ecotypes K2, K8 and K6 were efficient in the photosynthetic traits Phi2 and *F*_*v*_/*F*_*m*_ (Fig. 2A). Under WD, *F*_*v*_/*F*_*m*_ and Phi2 showed positive contribution in PC1 whereas, PC2 was associated with diversity among ecotypes due to the positive contribution of NT, DMY, Phi2 and *F*_*v*_/*F*_*m*_ (Fig. 2B). Ecotypes Kakamega, K15 and K23 inclined toward the direction of the NT and DM yield while K19, N3 and N1 clustered toward SPAD and PhiNPQ under WD conditions.

For the DTI, PC1 represented 75.25 % of the total variation among the ecotypes and was positively attributed to variation in all the indices except YSI. However, PC2 contributed 21.5 % of the total variation due to the positive contribution of Ys, GMP, YSI, YI and STI (Fig. 2C). Strong positive associations were observed among YI, Ys, STI, GMP, MP, Yp and TOL in the PCA biplot. Moreover, negative association between YSI and SSI was observed (Fig. 2C).

### Hierarchical cluster analysis

**Figure 3.**
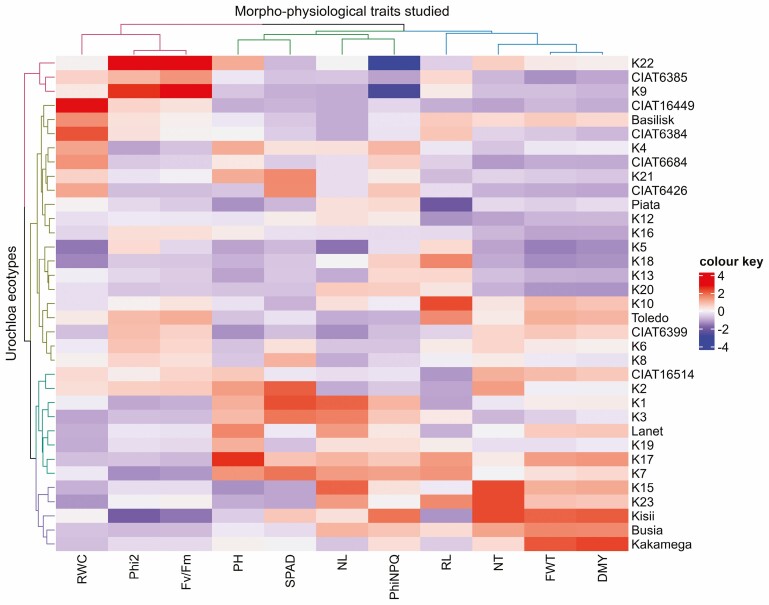
A heatmap with dendogram showing hierarchical clustering of 35 urochloa grass ecotypes and 11 studied traits under WD conditions. NT, number of tillers; PH, plant height; NL, number of leaves; RL, root length, FWT, fresh weight; DMY, dry matter yield; RWC, relative water content; Phi2, photosystem II photochemistry; PhiNPQ, non-photochemical quenching, SPAD, relative chlorophyll content; *F*_*v*_/*F*_*m*_, efficiency of photosystem II.

The 35 Urochloa ecotypes were grouped into five clusters: cluster I, cluster IIa, cluster IIb, cluster III and cluster IV, each comprising 3, 10, 9, 8 and 5 ecotypes, respectively. Each cluster had closely related ecotypes (Fig. 3).

Cluster I accounted for 8.6 % of all the studied ecotypes that had higher Phi2 and *F*_*v*_/*F*_*m*_ but recorded low values for all the other parameters and hence were considered susceptible to drought (Fig. 3, Table 3). Cluster II, comprised for 54.3 % of all ecotypes, which exhibited higher RWC and moderate chlorophyll content under WD and were considered moderately tolerant to drought stress (Table 3). Cluster IIb contained ecotypes with significantly longer roots but poor biomass yield. Cluster III had 37.1 % of the ecotypes, which were categorized as drought tolerant, with higher values of NT, PH, NL, Phi2 and SPAD under WD conditions. Cluster IV had mild tolerance to drought with higher Phi2 and *F*_*v*_/*F*_*m*_ but recorded low values for all the other parameters (Fig. 3 and Table 3).

**Table 3.** Mean ± SEM values for all traits of 35 *Urochloa* grass ecotypes under different clusters. NT, number of tillers; PH, plant height; NL, number of leaves; RL, root length, DMY, dry matter yield; RCW, relative water content; Phi2, photosystem II photochemistry; *F*_*v*_/*F*_*m*_, efficiency of photosystem II; PhiNPQ, non-photochemical quenching; SPAD, relative chlorophyll content.

Trait/Cluster	PH	NT	NL	RL	RWC	Phi2	PhiNPQ	SPAD	*F*_*v*_/*F*_*m*_	DMY
I	8.63 ± 1.53	3.13 ± 1.03	3.07 ± 0.18	52.00 ± 2.19	11.87 ± 0.76	0.31 ± 0.04	0.59 ± 0.05	2.25 ± 0.30	0.41 ± 0.04	0.66 ± 0.25
IIa	8.24 ± 0.64	2.48 ± 0.38	3.12 ± 0.01	50.62 ± 2.11	15.69 ± 1.66	0.18 ± 0.01	0.77 ± 0.01	5.59 ± 1.03	0.22 ± 0.01	1.61 ± 0.26
IIb	6.01 ± 0.31	3.31 ± 0.51	3.02 ± 0.16	56.96 ± 2.31	8.04 ± 1.07	0.19 ± 0.01	0.75 ± 0.15	4.10 ± 0.80	0.24 ± 0.01	0.87 ± 0.22
III	12.24 ± 0.57	4.18 ± 0.56	3.88 ± 0.22	50.55 ± 2.95	8.43 ± 1.10	0.16 ± 0.01	0.80 ± 0.02	8.98 ± 1.53	0.21 ± 0.02	1.33 ± 0.14
IV	6.68 ± 0.34	7.68 ± 0.86	3.88 ± 0.27	51.76 ± 3.60	6.88 ± 1.07	0.14 ± 0.02	0.82 ± 0.02	4.08 ± 1.26	0.19 ± 0.02	2.16 ± 0.21

Table 4 shows the mean ranking value for *Urochloa* grass ecotypes. Ecotypes K17, Kisii, Busia, K7 and Kakamega had the highest mean ranking values while ecotypes K13, CIAT6385, CIAT16449, K5 and K9 had the lowest mean ranking.

**Table 4.** Mean ranking values using PCA loading of morpho-physiological and yield traits for the 35 *Urochloa* grass ecotypes under WD conditions. Values are the mean of at least three independent replicates.

Ecotypes	PC1	PC2	PC3	PC4	Mean ranking value	Numerical rank
K17	2.89	0.74	1.00	1.88	159.94	1
Kisii	4.25	1.15	−0.10	−2.53	153.82	2
Busia	2.60	1.61	−0.85	−0.24	119.42	3
K7	3.02	−0.98	0.61	1.47	113.17	4
Kakamega	1.86	2.03	0.11	−1.02	107.16	5
K1	2.74	−1.39	1.78	0.49	98.43	6
Lanet	1.59	0.50	0.96	0.87	94.25	7
K23	1.27	2.43	−2.10	0.71	83.12	8
K15	2.01	1.27	−1.62	−0.47	75.91	9
K3	1.86	−1.56	0.68	1.82	61.60	10
CIAT16514	0.24	1.34	1.21	−1.04	46.66	11
K2	−0.24	0.52	2.72	−0.30	39.43	12
K10	0.29	1.52	−1.45	1.02	38.11	13
K19	0.55	−0.37	−0.27	1.06	19.57	14
K4	1.09	−1.59	0.84	−0.31	10.43	15
cv. Toledo	−1.10	2.09	−0.47	0.08	3.94	16
K21	0.07	−1.43	1.85	0.03	−4.84	17
K6	−0.87	0.93	−0.03	−0.20	−12.20	18
K22	−3.91	3.42	2.17	1.29	−15.59	19
cv.Basilisk	−0.62	0.85	−0.08	−1.11	−16.01	20
K8	−0.87	0.06	0.42	−0.39	−29.03	21
CIAT6399	−1.16	1.18	−0.79	−0.96	−36.72	22
K12	−0.39	−1.58	0.15	−0.07	−50.94	23
cv. Piata	−0.06	−1.03	−0.68	−1.60	−55.15	24
CIAT6426	0.04	−2.58	0.42	−0.84	−64.36	25
K16	−1.37	−1.00	−0.01	0.60	−67.74	26
K20	−0.18	−1.92	−1.42	0.47	−67.85	27
K18	−0.33	−1.70	−2.46	1.46	−71.98	28
CIAT6684	−0.62	−2.19	0.10	−0.80	−83.25	29
CIAT6384	−1.66	−0.89	0.13	−0.78	−89.45	30
K13	−0.82	−1.46	−1.60	−0.36	−92.26	31
CIAT6385	−2.89	−0.36	−0.01	0.56	−108.81	32
CIAT16449	−2.53	−1.36	0.36	0.32	−116.87	33
K5	−1.96	−1.30	−1.94	0.60	−124.42	34
K9	−4.80	1.50	0.40	0.76	−125.83	35

## Discussion

Forage breeders are constantly screening germplasm for selection of drought tolerant for utilization in advanced breeding programmes (Liu *et al.* 2015; Cheruiyot *et al.* 2018; Ajtahed *et al.* 2021). Greenhouse screening of germplasm for ability to withstand drought is an artificially induced method and has been reported to be an efficient way to identify drought tolerant forages (Fariaszewska *et al.* 2017). This is due to ease in the generation of uniform environmental conditions resulting in controlled plant growth (Ma *et al.* 2019). *Urochloa* is one of the important tropical forage grasses as it produces high tonnage of foliage biomass (Njarui *et al.* 2016). It is known to exhibit drought tolerance, although water availability still dictates the maximum yields achieved by *Urochloa* plants. This requires studies on cultivar-specific responses for the identification of *Urochloa* ecotypes with potential drought tolerance and high biomass and DMY. Drought stresses can occur at any point of plant growth and development, affecting major physiological processes in plants and thus reduce biomass yields (Luo *et al.* 2023). The present study evaluated the morpho-physiological and biomass yield responses to identify *Urochloa* grass ecotypes with drought adaptive traits.

The combined ANOVA analysis revealed significant variation among Urochloa ecotypes, WR and ecotype × WR interaction for the studied morpho-physiological and biomass yield traits. Similarly, significant genotype × moisture environment interaction was observed in in tall fescue grass (Ebrahimiyan *et al.* 2013), Brachiaria grass (Cheruiyot *et al.* 2018) and darum wheat (Chaouachi *et al.* 2023). The results indicate that grasses respond differently to soil moisture stress changing morpho-physiological traits.

The DMY reduced significantly when *Urochloa* grasses were exposed to WD conditions. Several studies have also reported similar results with grasses under water stress conditions. Zuffo *et al*. (2022) showed lower shoot dry matter content of *P. glaucum* under drought stress. In a field experiment, under drought stress conditions, the DMY of *Festuca*, *Festulolium* and *Lolium* grasses were significantly reduced (Fariasweska *et al.* 2020; Shariatipour *et al.* 2023) while Catunda *et al*. (2021) found that drought stress reduced the yields of tall fescue and lucerne. Similarly, a reduction in biomass production in drought stress has been observed in other *Urochloa* cultivars (Santos *et al.* 2013; Cardoso *et al.* 2015; Thaiana *et al.* 2020). However, high DMY were recorded for *Urochloa* ecotypes Kakamega, Kisii and Busia, which were similar to the cultivars Basilisk, Piata and Toledo used as controls under WD conditions. The high DMY obtained for the Kisii and Busia ecotypes could be linked to their high tillering ability while in Kakamega ecotype could be due to the broad leaves.

Morphological traits like tiller quantity, leaf size and leaf number directly influence fodder production capacity. For example, a significant correlation between NT and the shoot biomass in grasses has been reported (Zuffo *et al.* 2022). Drought stress significantly reduces photosynthesis and plant tillering causing significant losses in forage production (Staniak and Kocoń 2015; Fariasweska *et al.* 2020). WD reduced the NT, similar to that reported for other grasses (Hui *et al.* 2018). However, the NT in plants of ecotypes K7, K2, K6 and K22 did not change under WD conditions, an indication that the ecotypes gave priority to generating more tillers as a drought avoidance strategy. More tillers maximize plant use of available resources, such as water, capture more sunlight and thus increase photosynthesis efficiency. This is similar to tall wheatgrass (Borrajo *et al.* 2018) and perennial ryegrass (Turner *et al.* 2012) in which tillering reduction was not pronounced under water stress. Therefore, *Urochloa* ecotypes with the highest NT would be the most stable in WD conditions.

Ecotype K7, K10, K17, K18 and K23 had longer RL than other ecotypes under water stress. Longer RL improves plant water absorption, but elongated roots may hinder shoot growth as more photosynthetic products are translocated toward the roots (Zahid *et al.* 2021). The reduction in the NL under WD observed in this study is an adaptive strategy for *Urochloa* grasses to survive under drought stress conditions as reported by Staniak and Kocoń (2015).

Sufficient water is important in the plant’s life cycle. Leaf water status, estimated by leaf RWC, is crucial for assessing plant drought tolerance in water stress conditions. WD conditions reduced RWC in all the ecotypes examined. Similarly, Mastalerczuk and Borawska-Jarmułowicz (2021) observed a reduction in the RWC in drought stress in *Festulolium braunii*, *Lolium perenne* and *Festuca arundinacea*. Such decrease in RWC hinder grass growth under WD conditions (Wang *et al.* 2017; Faraszewska *et al.* 2020). However, accession CIAT16449 had a significantly higher RWC suggesting that different ecotypes of *Urochloa* grass have varying capacities to absorb soil moisture as well as distinct survival strategy in WD conditions (Mukami *et al.* 2019).

Drought stress significantly impacted chlorophyll content and efficiency of photosynthesis as measured by the MultispeQ device. Photosystem II photochemistry reduced in most of the ecotypes under WD conditions. The same findings were observed by Singh *et al.* (2022) and Akello *et al.* (2023). On the contrary, the K22 ecotype recorded increased Phi2 under water stress as was previously observed in ryegrass (Cielniak *et al.* 2006), *Arabidopsis* (Chen *et al.* 2016) and barley (Fernández-Calleja *et al.* 2020). Ecotype K22 may have transpired more often under water stress, relieving the electron pressure and allowing Phi2 to function more effectively (Fernández-Calleja *et al.* 2020).

A significant increase in PhiNPQ was observed with decreasing Phi2 under water stress. Ben-Jabeur *et al*. (2021) and Madumane *et al.* (2024) also recorded increasing PhiNPQ with drought stress. Thus, the plants preferred light-dependent dissipative processes involving PhiNPQ (Gómez *et al.* 2018). Drought-susceptible ecotypes CIAT6426 and CIAT6684 had the highest percentage increase in PhiNPQ in comparison to others under WD conditions. Ecotype K6 had slight increase in PhiNPQ implying less photosystem damage and downregulation of photosynthesis hence more drought tolerant. This is similar to a study by Singh *et al.* (2022) in which drought tolerant maize genotype increases PhiNPQ marginally in comparison to drought susceptible genotypes under drought stress. However, other authors observed a decrease in PhiNPQ with increasing drought stress (Singh *et al.* 2022; Akello *et al.* 2023). This could be due to differences in the plant species studied and the drought tolerance period applied in the experiments. A significant negative association between the increase in PhiNPQ and the decrease in Phi2 efficiency indicated that a larger proportion of the energy was thermally lost. This is due to PhiNPQ downregulation of photosynthesis by competing with photochemistry for absorbed energy (Singh *et al.* 2022). *Urochloa* grass ecotypes increased PhiNPQ either as a photoprotective response or non-regulated photoinhibition due to drought stress. Elevated PhiNPQ helps maintain photosynthesis through the dissipation of excess excitation energy as heat to protect photosynthetic apparatus (Phi2) from excessive exposure (Murchie and Ruban 2020; Ruban and Wilson 2021). Further studies are required to unlock the precise role of PhiNPQ under WD conditions in Urochloa grass ecotypes.

The *F*_*v*_/*F*_*m*_ measures linear electron transport rate and shows the overall plant photosynthetic capability (Shariatipour *et al.* 2023). A decline in *F*_*v*_/*F*_*m*_ was observed in all the ecotypes subjected to WD conditions. Similarly, Faraszewska *et al*. (2020) and Itam *et al.* (2024) previously reported a decrease in *F*_*v*_/*F*_*m*_ when *F. arundinacea* varieties and Kentucky bluegrass were subjected to soil moisture stress respectively. A decline in *F*_*v*_/*F*_*m*_ under drought indicates Photosystem II damage (Malan and Berner 2022; Shariatipour *et al.* 2023). The photosynthetic attributes showed genetic variation, with ecotype K22 and K9 having higher Phi2, PhiNPQ, SPAD and *F*_*v*_/*F*_*m*_. The high *F*_*v*_/*F*_*m*_ demonstrates improved net photosynthetic rate and biomass production under stress, which is a desirable trait to improve performance. Water stress did not affect K22 and K9 photochemical system, making them suitable ecotypes with better physiological adaptation under WD conditions. The increased *F*_*v*_/*F*_*m*_ was also associated with the efficiency of photosynthetic processes to use excess light energy, which was constrained by PhiNPQ under water stress (Sharma *et al.* 2015). Higher *F*_*v*_/*F*_*m*_ has also been associated with higher leaf temperature depression and higher percent of transpiration cooling rate leading to greater thermal stability of thylakoid membranes and lesser inhibition of Phi2 (Sharma *et al.* 2015).

Chlorophyll is the main pigment for photosynthesis and is most sensitive to water stress. Under drought stress conditions, relative chlorophyll content (SPAD) decreased, which is consistent with previous findings (Badr and Brüggemann 2020; Fernández-Calleja *et al.* 2020; De Souza *et al.* 2021; Mastalerczuk *et al.* 2022; Chaouachi *et al.* 2023; Shariatipour *et al.* 2023). This could be due to chlorophyll degradation in WD conditions. Improved productivity in water-limited situations has been linked to the plant’s capacity to maintain high chlorophyll concentrations (Zahid *et al.* 2021).

Significant positive correlation was observed between DMY, FWT, NT and RWC indicating that ecotypes with a high NT and the ability to maintain leaf water status (RWC) produced higher biomass yield (Zuffo *et al.* 2022). There was no correlation between the photosynthetic traits and yield components. This implies that exploiting photosynthetic features for indirect selection for yield under both WS and WD conditions is limited. Furthermore, compared to yield and morphological traits, photosynthetic attributes have distinct response pathways to drought, offering better insight into the physiological and mechanical elements of *Urochloa* grass’s tolerance to WD. When considered collectively, significant negative and positive relationships were discovered between several variables under WD, and these relationships can be used to uncover potential drought tolerant traits.

Comparing the drought tolerance levels of *Urochloa* grass ecotypes using one criterion or tolerance index is contradictory. Zuffo *et al*. (2022) observed that a single drought tolerance index did not precisely select maize genotypes for drought resistance. Ecotypes K17, Kisii, Busia, K7 and Kakamega were the most drought tolerant since they had the highest mean ranking values. The lowest mean rankings were observed in K13, CIAT6385, CIAT16449, K5 and K9 inferring susceptibility to drought.

Yield selection indices YI, GMP, STI, TOL and MP were useful in selection of *Urochloa* grass ecotypes that were high-yielding under both WD and WS conditions. DTI identified superior sorghum genotypes under well-watered and water stress environments (Abebe *et al.* 2020). The findings of this study also corroborated with Zuffo *et al*. (2022) who found STI, GMP and MP indices were useful in classification of forage grass cultivars to different drought tolerance levels. Ferede *et al*. (2020) and Mohammadi (2016) studied DTI in teff and wheat, respectively confirmed that high values of YI, STI, GMP, MP and YSI are the best indices for selection of high-yielding genotypes under both WS and WD conditions. With regard to YSI, ecotypes K13 and 16514 were the most desirable *Urochloa* ecotypes. The SSI can be utilized to identify ecotypes with relatively low Yp but high Ys (Ferede *et al.* 2020). Zahid *et al*. (2021) selected drought-tolerant cotton genotypes using SSI. Thus, breeders should also consider YSI and SSI to characterize breeding lines for drought tolerance.

The high-yielding ecotypes under both WD and WS (K17, Basilisk, K7, CIAT6399, Lanet, K23, K10, Kisii, Kakamega, K23 and Busia) were inclined to the direction of YI, Ys, STI, GMP, MP, Yp and TOL. The upper left quadrant of the PCA biplot had ecotypes (K13 and CIAT 16514) which are drought tolerant and characterized by the least percentage reduction in biomass yield under water stress as they clustered to the direction of YSI. Significant and negative association between YSI and SSI was recorded in this study (Zuffo *et al.* 2022). TOL was significant and positively associated with Ys, Yp, GMP and MP. However, TOL did not correlate with Ys in sorghum (Menezes *et al.* 2014) and soybean (Chiipanthenga 2020). Biplot reveals YI is optimal for evaluating ecotypes under drought stress, Kisii cultivar was the best suited to drought stress. SSI and YSI strongly negatively associate with Ys due to large obtuse angles. This result corroborates with other studies by Abebe *et al*. (2020) and Ferede *et al*. (2020). Furthermore, cultivars with high YSI cultivars yield less in non-stressed conditions and highest in stressed conditions (Ferede *et al.* 2020).

PCA biplot analysis shows *Urochloa* ecotypes near the origin and vector lines have superior breeding potential (Gedam *et al.* 2021). Under WD conditions, ecotypes K23, K15, Kakamega and Busia, were located near origin and along the FWT, DMY and NT vector line, can increase productivity in *Urochloa* breeding programmes.

## Conclusions

Morpho-physiological and yield traits varied among all the studied *Urochloa* grass ecotypes under both WS and WD conditions. Drought tolerance indicators (mean productivity, geometric mean productivity, tolerance index and stress tolerance index) were most effective in the identification of drought-tolerant ecotypes. The study revealed variation among *Urochloa* grass ecotypes and provided a theoretical basis for improving tolerance of *Urochloa* grass ecotypes to drought stress. Ecotypes K17, Kisii, Busia, K7 and Kakamega depicted greater drought adaptation with higher biomass yield and mean ranking values under WD. The five drought tolerant ecotypes identified need to be tested further under field for sustainable forage grass production under drought stress conditions. These ecotypes with drought-adaptive traits could be utilized in breeding programs to develop high-yielding and drought-tolerant varieties. Further studies could elucidate the biochemical and molecular mechanisms behind tolerance to drought stress to achieve tangible progress in *Urochloa* grass breeding programmes.

## Supporting Information

The following additional information is available in the online version of this article –

**Table S1.** List of *Urochloa* ecotypes used in the study.

**Table S2.** Mean squares values for the morpho-physiological and yield traits through Generalized Linear Model Analysis of Variance. *** and ** significant at *P* < 0.001 and *P* < 0.01, respectively. PH, plant height; NT, number of tillers; NL, number of leaves; RL, root length, DMY, dry matter yield; FWT, fresh weight; RCW, relative water content; Phi2, photosystem II photochemistry; PhiNPQ, non-photochemical quenching; SPAD, relative chlorophyll content; *F*_*v*_/*F*_*m*_, efficiency for photosystem II.

**Table S3.** Mean biomass yield for each ecotype under different WR. CV, coefficient of variation; LSD, least significance difference. Values expressed as Mean ± SEM (*n* = 5).

**Table S4.** Correlation coefficients (*r*) between Biomass yield of Urochloa ecotypes under non-stressed and stressed conditions and among selected indices. Symbols ***, ** and *shows significant at 0.001, 0.01 and 0.05, respectively. NS, non-significant; MP, mean productivity; GMP, geometric mean productivity; TOL, tolerance index; YSI, yield stability index; YI, yield index; Yp, mean Biomass yield under WS; Ys, mean Biomass yield under WD; STI, stress tolerance index; SSI, stress susceptible index.

plae034_suppl_Supplementary_Tables

## Data Availability

All data generated and used is included in this article and supplementary file.

## References

[CIT0001] Abebe T, Belay G, Tadesse T, Keneni G. 2020. Selection efficiency of yield based drought tolerance indices to identify superior sorghum [*Sorghum bicolor* (L.) Moench] genotypes under two-contrasting environments. African Journal of Agricultural Research 15:379–392.

[CIT0002] Affoh R, Zheng H, Dangui K, Dissani BM. 2022. The impact of climate variability and change on food security in sub-saharan Africa: perspective from panel data analysis. Sustainability 14:759.

[CIT0003] Aghaie P, Tafreshi SAH, Ebrahimi MA, Haerinasab M. 2018. Tolerance evaluation and clustering of fourteen tomato cultivars grown under mild and severe drought conditions. Scientia Horticulturae 232:1–12.

[CIT0004] Ajtahed SS, Rezaei A, Hosseini Tafreshi SA. 2021. Identifying superior drought-tolerant Bermudagrass accessions and their defensive responses to mild and severe drought conditions. Euphytica 217:1–21.

[CIT0005] Akello M, Nyaboga EN, Badji A, Rubaihayo P. 2023. Deciphering the morpho-physiological and biochemical responses in *Lablab purpureus* (L.) sweet seedlings to water stress. South African Journal of Botany 162:412–424.

[CIT0006] Badr A, Brüggemann W. 2020. Comparative analysis of drought stress response of maize genotypes using chlorophyll fluorescence measurements and leaf relative water content. Photosynthetica 58:638–645.

[CIT0007] Ben-Jabeur M, Gracia-Romero A, López-Cristoffanini C, Vicente R, Kthiri Z, Kefauver SC, Hamada W. 2021. The promising MultispeQ device for tracing the effect of seed coating with biostimulants on growth promotion, photosynthetic state and water–nutrient stress tolerance in durum wheat. Euro-Mediterranean Journal for Environmental Integration 6:8.

[CIT0008] Borrajo CI, Sánchez-Moreiras AM, Reigosa MJ. 2018. Morpho-physiological responses of tall wheatgrass populations to different levels of water stress. PLoS One 13:e0209281. 30557312 10.1371/journal.pone.0209281 PMC6296543

[CIT0009] Cardoso JA, Pineda M, Jiménez JDLC, Vergara MF, Rao IM. 2015. Contrasting strategies to cope with drought conditions by two tropical forage C4 grasses. AoB Plants 7:plv107. 26333827 10.1093/aobpla/plv107 PMC4595746

[CIT0010] Catunda KL, Churchill AC, Zhang H, Power SA, Moore BD. 2021. Short‐term drought is a stronger driver of plant morphology and nutritional composition than warming in two common pasture species. Journal of Agronomy and Crop Science 208:841–852.

[CIT0011] Chaouachi L, Marín-Sanz M, Kthiri Z, Boukef S, Harbaoui K, Barro F, Karmous C. 2023. The opportunity of using durum wheat landraces to tolerate drought stress: screening morpho-physiological components. AoB Plants 15:plad022. 37228421 10.1093/aobpla/plad022 PMC10205476

[CIT0012] Chen D, Wang S, Cao B, Cao D, Leng G, Li H, Deng X. 2016. Genotypic variation in growth and physiological response to drought stress and re-watering reveals the critical role of recovery in drought adaptation in maize seedlings. Frontiers in Plant Science 6:1241. 26793218 10.3389/fpls.2015.01241 PMC4709455

[CIT0013] Cheruiyot D, Aura Odhiambo Midega C, Van den Berg J, Pickett JA, Rahman Khan Z. 2018. Genotypic responses of brachiaria grass (*Brachiaria* spp.) accessions to drought stress. Journal of Agronomy 17:136–146.

[CIT0014] Chiipanthenga MK. 2020. Drought tolerance in Malawian soybean (Glycine Max L.) germplasm. Doctoral dissertation, University of the Free State.

[CIT0015] Cielniak JK, Filek W, Cielniak JBK. 2006. The effect of drought stress on chlorophyll fluorescence in Lolium-Festuca hybrids. Acta Physiologiae Plantarum 28:149–158.

[CIT0016] De Souza EMB, da Rocha WSD, de Avila Soares N, Martins CE, de Souza Sobrinho F, de Almeida MIV, Moreira GR. 2021. Water deficit tolerance in genotypes of *Urochloa* spp.: water deficit in *Urochloa* spp. Revista de Ciências Agrárias 44:2–3

[CIT0017] Dinar A, Tieu A, Huynh H. 2019. Water scarcity impacts on global food production. Global Food Security 23:212–226.

[CIT0018] Djikeng A, Rao IM, Njarui D, Mutimura M, Caradus J, Ghimire SR, Johnson L, Cardoso JA, Ahonsi M, Kelemu S; icipe ? African Insect Science for Food and Health, Nairobi, Kenya. www.icipe.org. 2014. Climate-smart *Brachiaria* grasses for improving livestock production in East Africa. Tropical Grasslands—Forrajes Tropicales 2:38–39.

[CIT0019] Ebrahimiyan M, Majidi MM, Mirlohi A. 2013. Genotypic variation and selection of traits related to forage yield in tall fescue under irrigated and drought stress environments. Grass and Forage Science 68:59–71.

[CIT0020] Fariaszewska A, Aper J, Van Huylenbroeck J, Baert J, De Riek J, Staniak M, Pecio L. 2017. Mild drought stress‐induced changes in yield, physiological processes and chemical composition in Festuca, Lolium and Festulolium. Journal of Agronomy and Crop Science 203:103–116.

[CIT0021] Fariaszewska A, Aper J, Van Huylenbroeck J, De Swaef T, Baert J, Pecio L. 2020. Physiological and biochemical responses of forage grass varieties to mild drought stress under field conditions. International Journal of Plant Production 14:335–353.

[CIT0022] Ferede B, Mekbib F, Assefa K, Chanyalew S, Abraha E, Tadele Z. 2020. Evaluation of drought tolerance in Tef [*Eragrostis tef* (Zucc.) trotter] genotypes using drought tolerance indices. Journal of Crop Science and Biotechnology 23:107–115.

[CIT0023] Fernández-Calleja M, Monteagudo A, Casas AM, Boutin C, Pin PA, Morales F, Igartua E. 2020. Rapid on-site phenotyping via field fluorimeter detects differences in photosynthetic performance in a hybrid—parent barley germplasm set. Sensors (Basel, Switzerland) 20:1486. 32182722 10.3390/s20051486 PMC7085516

[CIT0024] Ferreira RCU, Costa Lima Moraes AD, Chiari L, Simeão RM, Vigna BBZ, de Souza AP. 2021. An overview of the genetics and genomics of the urochloa species most commonly used in pastures. Frontiers in Plant Science 12:770461. 34966402 10.3389/fpls.2021.770461 PMC8710810

[CIT0072] Fischer RA, Maurer R. 1978. Drought resistance in spring wheat cultivars. I. Grain yield responses. *Australian Journal of Agricultural Research* 29:897–912.

[CIT0025] Gedam PA, Thangasamy A, Shirsat DV, Ghosh S, Bhagat KP, Sogam OA, Singh M. 2021. Screening of onion (*Allium cepa* L.) genotypes for drought tolerance using physiological and yield based indices through multivariate analysis. Frontiers in Plant Science 12:600371. 33633759 10.3389/fpls.2021.600371 PMC7900547

[CIT0026] GoK, Government of Kenya. (2019). *Draft National livestock policy, 2019*. https://repository.kippra.or.ke/bitstream/handle/123456789/483/Draft-reviewed-National-Livestock-Policy-February-2019.pdf (accessed 5 May 2024).

[CIT0027] Gómez R, Carrillo N, Morelli MP, Tula S, Shahinnia F, Hajirezaei MR, Lodeyro AF. 2018. Faster photosynthetic induction in tobacco by expressing cyanobacterial flavodiiron proteins in chloroplasts. Photosynthesis Research 136:129–138. 29022124 10.1007/s11120-017-0449-9

[CIT0028] Hui D, Yu CL, Deng Q, Dzantor EK, Zhou S, Dennis S, Luo Y. 2018. Effects of precipitation changes on switchgrass photosynthesis, growth, and biomass: a mesocosm experiment. PLoS One 13:e0192555. 29420600 10.1371/journal.pone.0192555 PMC5805322

[CIT0029] Itam M, Hall D, Kramer D, Merewitz E. 2024. Early detection of Kentucky bluegrass and perennial ryegrass responses to drought stress by measuring chlorophyll fluorescence parameters. Crop Science 64:1015–1026.

[CIT0030] Kassambara A., Mundt F. 2017. Factoextra: extract and visualize the results of multivariate data analyses, R package version 1.0. 5.999. R Core Team. https://CRAN.R-project.org/package=factoextra.

[CIT0031] Kuhlgert S, Austic G, Zegarac R, Osei-Bonsu I, Hoh D, Chilvers MI, Kramer DM. 2016. MultispeQ Beta: a tool for large-scale plant phenotyping connected to the open PhotosynQ network. Royal Society Open Science 3:160592. 27853580 10.1098/rsos.160592 PMC5099005

[CIT0032] Liu Y, Zhang X, Tran H, Shan L, Kim J, Childs K, Zhao B. 2015. Assessment of drought tolerance of 49 switchgrass (*Panicum virgatum*) genotypes using physiological and morphological parameters. Biotechnology for Biofuels 8:1–18. 26396590 10.1186/s13068-015-0342-8 PMC4578271

[CIT0033] Luo Q, Xie H, Chen Z, Ma Y, Yang H, Yang B, Ma Y. 2023. Morphology, photosynthetic physiology and biochemistry of nine herbaceous plants under water stress. Frontiers in Plant Science 14:1147208–1147208. 37063188 10.3389/fpls.2023.1147208 PMC10098446

[CIT0034] Ma D, Carpenter N, Amatya S, Maki H, Wang L, Zhang L, Jin J. 2019. Removal of greenhouse microclimate heterogeneity with conveyor system for indoor phenotyping. Computers and Electronics in Agriculture 166:104979.

[CIT0073] Madumane K, Sewelo LT, Nkane MN, Batlang U, Malambane G. 2024. Morphological, physiological, and molecular stomatal responses in local watermelon landraces as drought tolerance mechanisms. *Horticulturae* 10:123.

[CIT0035] Majidi MM, Hosseini B, Barati M, Mirlohi A, Araghi B. 2016. Simultaneous selection for seed and forage production in cocks-foot: application of drought tolerance and susceptibility indices. Grass and Forage Science 72:441–453.

[CIT0036] Malan C, Berner JM. 2022. Comparative PSII photochemistry of quinoa and maize under mild to severe drought stress. Photosynthetica 60:362–371. 10.32615/ps.2022.022 PMC11558597 39650103

[CIT0070] Marchin RM, Ossola A, Leishman MR, Ellsworth DS. 2020. A simple method for simulating drought effects on plants. *Frontiers in Plant Science* 10:493655. 10.3389/fpls.2019.01715 PMC6985571 32038685

[CIT0037] Martin R, Linstädter A, Frank K, Müller B. 2016. Livelihood security in face of drought – assessing the vulnerability of pastoral households. Environmental Modelling and Software 75:414–423.

[CIT0038] Mastalerczuk G, Borawska-Jarmułowicz B. 2021. Physiological and morphometric response of forage grass species and their biomass distribution depending on the term and frequency of water deficiency. Agronomy 11:2471.

[CIT0039] Mastalerczuk G, Borawska-Jarmułowicz B, Darkalt A. 2022. Changes in the physiological and morphometric characteristics and biomass distribution of forage grasses growing under conditions of drought and silicon application. Plants (Basel, Switzerland) 12:16. 36616145 10.3390/plants12010016 PMC9823582

[CIT0040] Menezes CBD, Ticona-Benavente CA, Tardin FD, Cardoso MJ, Bastos EA, Nogueira DW, Portugal AF, Santos CV, Schaffert RE. 2014. Selection indices to identify drought-tolerant grain sorghum cultivars. 10.4238/2014.November.27.9 25501191

[CIT0041] Mohammadi R. 2016. Efficiency of yield-based drought tolerance indices to identify tolerant genotypes in durum wheat. Euphytica 211:71–89.

[CIT0042] Mukami A, Ngetich A, Mweu C, Oduor RO, Muthangya M, Mbinda WM. 2019. Differential characterization of physiological and biochemical responses during drought stress in finger millet varieties. Physiology and Molecular Biology of Plants : an International Journal of Functional Plant Biology 25:837–846. 31402813 10.1007/s12298-019-00679-z PMC6656826

[CIT0043] Murchie EH, Ruban AV. 2020. Dynamic non‐photochemical quenching in plants: from molecular mechanism to productivity. The Plant Journal 101:885–896. 31686424 10.1111/tpj.14601

[CIT0044] Mutimura M., Ghimire S. 2021. Brachiaria grass for sustainable livestock production in Rwanda under climate change. In Leal Filho W, Luetz J, Ayal D, eds. Handbook of climate change management: research, leadership, transformation. Cham: Springer International Publishing, 195–211.

[CIT0045] Naghavi MR, Aboughadareh AP, Khalili M. 2013. Evaluation of drought tolerance indices for screening some of corn (*Zea mays* L.) cultivars under environmental conditions. Notulae Scientia Biologicae 5:388–393.

[CIT0046] Nandakumar S, Pipil H, Ray S, Haritash AK. 2019. Removal of phosphorous and nitrogen from wastewater in Brachiaria-based constructed wetland. Chemosphere 233:216–222. 31173959 10.1016/j.chemosphere.2019.05.240

[CIT0047] Negawo AT, Teshome A, Kumar A, Hanson J, Jones CS. 2017. Opportunities for Napier grass (*Pennisetum purpureum*) improvement using molecular genetics. Agronomy 7:28–28.

[CIT0049] Njarui DMG, Gatheru M, Ghimire SR, Mureithi JG. 2016. Effects of seasons and cutting intervals on productivity and nutritive value of Brachiaria grass cultivars in semi-arid eastern Kenya. Climate Smart Brachiaria Grasses for Improving Livestock Production in East Africa–Kenya Experience 1:46–48.

[CIT0048] Njarui DMG, Gatheru M, Ghimire SR. 2020. Brachiaria grass for climate resilient and sustainable livestock production in Kenya. African Handbook of Climate Change Adaptation: 1–22.

[CIT0050] OECD, Organisation for Economic Cooperation and Development. 2018. OECD-FAO Agricultural Outlook 2018-2027. OECD Agriculture statistics (database). 10.1787/agr-data-en.

[CIT0051] Poorter H, Bühler J, van Dusschoten D, Climent J, Postma JA. 2012. Pot size matters: a meta-analysis of the effects of rooting volume on plant growth. Functional Plant Biology 39:839–850. 32480834 10.1071/FP12049

[CIT0052] Putranto DH. 2018. Stomatal conductance and chlorophyll fluorescence of oil palm under field conditions. Wageningen: Wageningen University.

[CIT0071] R Core Team. 2021. R: a language and environment for statistical computing. R Foundation for Statistical Computing. https://www.R-project.org/.

[CIT0053] Rohde MM. 2023. Floods and droughts are intensifying globally. Nature Water 1:226–227.

[CIT0054] Ruban AV, Wilson S. 2021. The mechanism of non-photochemical quenching in plants: localization and driving forces. Plant and Cell Physiology 62:1063–1072. 33351147 10.1093/pcp/pcaa155

[CIT0055] Santos PM, Cruz PG, Araujo LC, Pezzopane JRM, Valle CB, Pezzopane CG. 2013. Response mechanisms of *Brachiaria brizantha* cultivars to water déficit stress. Revista Brasileira de Zootecnia 42:773.

[CIT0056] Schneider F, Tarawali S. 2021. Sustainable Development Goals and livestock systems. Revue Scientifique et Technique (International Office of Epizootics) 40:585–595. 34542093 10.20506/rst.40.2.3247

[CIT0057] Shariatipour N, Shams Z, Heidari B, Richards C. 2023. Genetic variation and response to selection of photosynthetic and forage characteristics in Kentucky bluegrass (*Poa pratensis* L.) ecotypes under drought conditions. Frontiers in Plant Science 14:1239860. 38023869 10.3389/fpls.2023.1239860 PMC10667697

[CIT0058] Sharma DK, Andersen SB, Ottosen CO, Rosenqvist E. 2015. Wheat cultivars selected for high *F*_*v*_/*F*_*m*_ under heat stress maintain high photosynthesis, total chlorophyll, stomatal conductance, transpiration and dry matter. Physiologia Plantarum 153:284–298. 24962705 10.1111/ppl.12245

[CIT0059] Singh GM, Goldberg S, Schaefer D, Zhang F, Sharma S, Mishra VK, Xu J. 2022. Biochemical, gas exchange, and chlorophyll fluorescence analysis of maize genotypes under drought stress reveals important insights into their interaction and homeostasis. Photosynthetica 60:376–388. 10.32615/ps.2022.024 PMC11558602 39650104

[CIT0060] Staniak M. 2016. The impact of drought stress on the yields and food value of selected forage grasses. Acta Agrobotanica 69:1663–1674. doi:10.23986/afsci.73282

[CIT0061] Staniak M, Kocoń A. 2015. Forage grasses under drought stress in conditions of Poland. Acta Physiologiae Plantarum 37:1–10.

[CIT0062] Staver AC, Wigley-Coetsee C, Botha J. 2019. Grazer movements exacerbate grass declines during drought in an African savanna. Journal of Ecology 107:1482–1491.

[CIT0063] Thaiana Rueda da Silva C, Bonfim-Silva EM, De Araujo da Silva TJ, Alves Rodrigues Pinheiro E, Vieira José J, Pereira Freire Ferraz A. 2020. Yield component responses of the *Brachiaria brizantha* forage grass to soil water availability in the Brazilian Cerrado. Agriculture 10:13.

[CIT0064] Turner LR, Holloway‐Phillips MM, Rawnsley RP, Donaghy DJ, Pembleton KG. 2012. The morphological and physiological responses of perennial ryegrass (*Lolium perenne* L.), cocksfoot (*Dactylis glomerata* L.) and tall fescue (*Festuca arundinacea* Schreb.; syn. *Schedonorus phoenix* Scop.) to variable water availability. Grass and Forage Science 67:507–518.

[CIT0065] Van Thanh Ho T, Dang MP, Tu TL, Thien TH, Bach LG. 2020. Assessing the ability to treat industrial wastewater by constructed wetland model using the Brachiaria mutica. Waste and Biomass Valorization 11:5615–5626.

[CIT0066] Wang J, Burgess P, Bonos SA, Meyer WA, Huang B. 2017. Differential physiological responses and genetic variations in fine fescue species for heat and drought stress. Journal of the American Society for Horticultural Science 142:367–375.

[CIT0067] Zahid Z, Khan MKR, Hameed A, Akhtar M, Ditta A, Hassan HM, Farid G. 2021. Dissection of drought tolerance in upland cotton through morpho-physiological and biochemical traits at seedling stage. Frontiers in Plant Science 12:627107. 33777067 10.3389/fpls.2021.627107 PMC7994611

[CIT0068] Zhang H, Zhu J, Gong Z, Zhu JK. 2022. Abiotic stress responses in plants. Nature Reviews Genetics 23:104–119. 10.1038/s41576-021-00413-0 34561623

[CIT0069] Zuffo AM, Steiner F, Aguilera JG, Ratke RF, Barrozo LM, Mezzomo R, Ancca SM. 2022. Selected indices to identify water-stress-tolerant tropical forage grasses. Plants 11:2444. 36145845 10.3390/plants11182444 PMC9504478

