# Correction to: Morpho-physiological and yield traits for selection of drought tolerant *Urochloa* grass ecotypes

**DOI:** 10.1093/aobpla/plae052

**Published:** 2024-10-05

This is a correction to: Celestine Anyango Ochola, Mathew Pierro Ngugi, Evans N Nyaboga, Donald M G Njarui, Morpho-physiological and yield traits for selection of drought tolerant *Urochloa* grass ecotypes, *AoB PLANTS*, Volume 16, Issue 3, June 2024, plae034, https://doi.org/10.1093/aobpla/plae034

The following changes have been made to the originally published manuscript. In the section **Measurement of physiological traits**, an abbreviation was added for “turgid weight (TW)” and the corresponding formula altered to read:


$$\begin{array}{l} Relative\;water\;content=\left(\frac{FWT-DMY}{TW-DMY}\right)\times 100\% \end{array}$$

instead of

“*Relative water content (RWC)* =”.

