# Supplementary material for: Morpho-physiological and yield traits for selection of drought tolerant Urochloa grass ecotypes
Source: AoB Plants. 2024 Jun 6;16(3):plae034. doi: 10.1093/aobpla/plae034 (PMC11212069; doi:10.1093/aobpla/plae034)
Supplement: plae034_suppl_Supplementary_Tables [file plae034_suppl_supplementary_tables.pdf]

Supplementary data

## Table S1: List of *Urochloa* ecotypes used in the study

| Accessions No | Species | Location | Latitude | Longitude | Altitude (m asl) |
|---|---|---|---|---|---|
| K1 | ***Urochloa*** **spp** | Makueni | 1.71789E | 37.57585S | 1211 |
| K2 | ***Urochloa*** **spp** | Makueni | 1.73533E | 37.57272S | 1214 |
| K3 | ***Urochloa*** **spp** | Makueni | 1.78682E | 37.55466S | 1185 |
| K4 | ***Urochloa*** **spp** | Makueni | 1.77815E | 37.55948S | 1178 |
| K5 | ***Urochloa*** **spp** | Makueni | 1.73861E | 37.54112S | 1338 |
| K6 | ***Urochloa*** **spp** | Makueni | 1.74028E | 37.53921S | 1355 |
| K7 | ***Urochloa*** **spp** | Makueni | 1.75530E | 37.53325S | 1254 |
| K8 | ***Urochloa*** **spp** | Makueni | 1.80072E | 37.50563S | 1551 |
| K9 | ***Urochloa*** **spp** | Makueni | 1.80538E | 37.119035S | 1348 |
| K10 | ***Urochloa*** **spp** | Makueni | 1.80507E | 37.49308S | 1414 |
| K12 | ***Urochloa*** **spp** | Makueni | 1.79295E | 37.49523S | 1340 |
| K13 | ***Urochloa*** **spp** | Makueni | Unknown | Unknown | Unknown |
| K15 | ***Urochloa*** **spp** | Machakos | 1.29872E | 37.3450S | 1500 |
| K16 | ***Urochloa*** **spp** | Makueni | Unknown | Unknown | Unknown |
| K17 | ***Urochloa*** **spp** | Kitui | 1.37444E | 37.5901S | 1160 |
| K18 | ***Urochloa*** **spp** | Makueni | 1.76033E | 37.45837S | 1301 |
| K19 | ***Urochloa*** **spp** | Makueni | 1.76033E | 37.45837S | 1301 |
| K20 | ***Urochloa*** **spp** | Makueni | 1.75760E | 37.46780S | 1282 |
| K21 | ***Urochloa*** **spp** | Makueni | 1.78682E | 37.55466S | 1185 |
| K22 | ***Urochloa*** **spp** | Makueni | 1.74806E | 37.45452S | 1314 |
| K23 | ***Urochloa*** **spp** | Nairobi | 1.15377E | 36.46414S | 1789 |
| Busia | ***Urochloa spp*** | Busia | Unknown | Unknown | Unknown |
| Lanet | ***Urochloa spp*** | Lanet | Unknown | Unknown | Unknown |
| Kisii | ***Urochloa spp*** | Kisii | Unknown | Unknown | Unknown |
| Kakamega | ***Urochloa spp*** | Kakamega | Unknown | Unknown | Unknown |
| CIAT16514 | ***U. jubata*** | Trans Nzoia | 1.1167E | 35.0667S | 1920 |
| CIAT6385 | ***U. brizantha*** | Rift valley | 0.6012E | 35.5333S | 2120 |
| CIAT6384 | ***U. brizantha*** | Rift valley | -0.0667E | 34.6833S | 1400 |
| CIAT6399 | ***U. brizantha*** | Rift valley | Unknown | Unknown | 2130 |
| CIAT6426 | ***U. brizantha*** | Rift valley | 0.5833E | 35.3667S | 2300 |
| CIAT6684 | ***U. brizantha*** | Rift valley | 0.3501E | 34.8167S | 1606 |
| CIAT16449 | ***U. brizantha*** | Rift valley | - | - | - |
| Basilisk | ***U. decumbens*** | Machakos | 1.5833E | 37.2333S | 1600 |
| Toledo | ***U. brizantha*** | Machakos | 1.5833E | 37.2333S | 1600 |
| Piata | ***U. brizantha*** | Machakos | 1.5833E | 37.2333S | 1600 |

**Table S2:** Mean squares values for the morpho-physiological and yield traits through Generalized Linear Model Analysis of Variance

| Source of variation | DF | PH | NT | NL | RL | RWC | Phi2 | PhiNPQ | SPAD | Fv/Fm | FWT | DMY |
|---|---|---|---|---|---|---|---|---|---|---|---|---|
| Ecotypes (Eco) | 34 | 161.23*** | 52.37*** | 2.99*** | 751.70*** | 409.00*** | 0.054*** | 0.040*** | 69.1*** | 0.029*** | 86.34*** | 17.62*** |
| Water Regime (WR) | 1 | 3741.2*** | 132.07*** | 177.15*** | 49825.6*** | 356290*** | 2.695*** | 2.302*** | 87921.5*** | 2.806*** | 3655.89*** | 403.5*** |
| ECo X WR | 34 | 50.06*** | 6.97*** | 1.78*** | 892.2*** | 242*** | 0.040*** | 0.030*** | 96.8*** | 0.016** | 56.29*** | 6.64*** |
| Error | 280 | 0.38 | 0.64 | 0.79 | 107.2 | 20 | 0.006 | 0.011 | 5.5 | 0.009 | 6.91 | 0.27 |
| Total | 349 | | | | | | | | | | | |

***, ** Significant at P<0.001, P<0.01respectively; PH, Plant Height; NT, Number of Tillers; NL, Number of Leaves; RL, Root Length, DMY, Dry matter yield; FWT, Fresh weight; RCW, Relative Water Content; Phi2, photosystem II photochemistry; PhiNPQ, non-photochemical quenching; SPAD, Relative chlorophyll content; Fv/Fm, efficiency for photosystem II

**Table S3:** Mean biomass yield for each ecotype under different water regimes. CV, Coefficient of Variation; LSD, Least Significance Difference. Values expressed as Mean±SEM(n=5)

| | Fresh weight | | Dry weight | |
|---|---|---|---|---|
| Ecotypes | WS | WD | WS | WD |
| CIAT16449 | 1.88±1.07 | 0.65±0.30 | 1.49±0.22 | 0.40±0.19 |
| CIAT16514 | 5.20±1.46 | 1.86±0.18 | 2.57±0.19 | 1.56±0.16 |
| CIAT6384 | 3.84±1.09 | 0.80±0.18 | 1.52±0.30 | 0.51±0.13 |
| CIAT6385 | 1.75±0.94 | 0.30±0.07 | 0.70±0.29 | 0.35±0.17 |
| CIAT6399 | 14.56±2.20 | 1.75±0.39 | 5.25±0.40 | 1.44±0.36 |
| CIAT6426 | 1.83±0.34 | 0.52±0.16 | 0.93±0.13 | 0.35±0.13 |
| CIAT6684 | 2.95±1.59 | 0.53±0.16 | 1.09±0.13 | 0.38±0.15 |
| BSK | 20.98±1.91 | 1.70±0.27 | 9.12±0.28 | 1.39±0.24 |
| Busia | 9.83±0.86 | 2.36±0.09 | 3.86±0.23 | 2.16±0.08 |
| K1 | 7.74±1.47 | 1.33±0.24 | 3.51±0.19 | 1.18±0.14 |
| K10 | 11.84±3.68 | 1.88±0.77 | 4.99±0.26 | 1.61±0.15 |
| K12 | 3.57±1.68 | 0.60±0.16 | 2.64±0.09 | 0.50±0.15 |
| K13 | 1.12±0.17 | 0.56±0.11 | 0.41±0.11 | 0.41±0.10 |
| K15 | 6.11±1.30 | 2.00±0.27 | 2.94±0.24 | 1.87±0.25 |
| K16 | 3.00±1.49 | 0.45±0.11 | 1.57±0.26 | 0.34±0.08 |
| K17 | 13.66±1.43 | 2.16±0.10 | 4.86±0.38 | 2.03±0.13 |
| K18 | 3.47±1.76 | 0.27±0.04 | 1.41±0.13 | 0.19±0.06 |
| K19 | 11.83±1.39 | 1.02±0.12 | 5.23±0.38 | 0.97±0.10 |
| K2 | 6.68±0.90 | 1.17±0.09 | 2.55±0.23 | 1.03±0.09 |
| K20 | 1.10±0.22 | 0.34±0.08 | 0.41±0.09 | 0.21±0.06 |
| K21 | 3.83±1.59 | 0.82±0.14 | 1.43±0.16 | 0.64±0.13 |
| K22 | 7.98±2.49 | 1.35±0.32 | 3.18±0.54 | 1.14±0.29 |
| K23 | 11.05±1.30 | 1.80±0.32 | 3.92±0.24 | 1.57±0.32 |
| K3 | 4.00±0.33 | 0.86±0.07 | 1.42±0.17 | 0.92±0.15 |
| K4 | 6.61±2.51 | 1.08±0.15 | 3.76±0.32 | 0.98±0.13 |
| K5 | 2.67±0.38 | 0.16±0.05 | 1.10±0.06 | 0.14±0.04 |
| K6 | 7.70±02.54 | 1.350.26 | 3.82±0.45 | 1.13±0.10 |
| K7 | 14.69±0.68 | 1.47±0.29 | 5.39±0.11 | 1.39±0.30 |
| K8 | 7.06±1.38 | 1.12±0.40 | 3.00±0.38 | 0.90±0.37 |
| K9 | 3.80±1.33 | 0.70±0.22 | 1.58±0.22 | 0.48±0.20 |
| Kak | 16.71±1.39 | 2.77±0.40 | 5.89±0.17 | 2.70±0.17 |
| Kisii | 14.56±2.15 | 2.66±0.39 | 5.51±0.19 | 2.52±0.16 |
| Lanet | 10.66±1.32 | 1.71±0.11 | 5.33±0.23 | 1.57±0.11 |
| Piata | 7.62±1.44 | 0.86±0.21 | 3.13±0.25 | 0.83±0.21 |
| Xaraes | 17.31±2.94 | 1.99±0.55 | 7.20±0.52 | 1.76±0.30 |
| Mean | 7.69±0.47 | 1.23±0.07 | 3.22±0.16 | 1.07±0.06 |
| LSD | 0.90 | 0.12 | 0.16 | 0.06 |
| %CV | 69.09 | 57.93 | 34.42 | 72.38 |

**Table S4:** Correlation coefficients (r) between Biomass yield of Urochloa ecotypes under non-stressed and stressed conditions and among selected indices.***, **, * = significant at 0.001, 0.01 and 0.05 respectively, NS = non-significant, MP= mean productivity, GMP = geometric mean productivity, TOL = tolerance index, YSI = yield stability index, YI = yield index, Yp = mean Biomass yield under water sufficient, Ys = mean Biomass yield under water deficit, STI = stress tolerance index, SSI = stress susceptible index.

| | Yp | Ys | MP | GMP | TOL | YSI | YI | SSI | STI |
|---|---|---|---|---|---|---|---|---|---|
| Yp | 1.00 | | | | | | | | |
| Ys | 0.84** | 1.00 | | | | | | | |
| MP | 1.00*** | 0.87** | 1.00 | | | | | | |
| GMP | 0.97*** | 0.94*** | 0.99*** | 1.00 | | | | | |
| TOL | 1.00*** | 0.81** | 0.99*** | 0.96*** | 1.00 | | | | |
| YSI | -0.74* | -0.29NS | -0.70* | -0.59NS | -0.78** | 1.00 | | | |
| YI | 0.84** | 1.00*** | 0.87** | 0.94*** | 0.81** | -0.29NS | 1.00 | | |
| SSI | 0.74* | 0.29NS | 0.70* | 0.59NS | 0.78** | -1.00*** | 0.29NS | 1.00 | |
| STI | 0.97*** | 0.94*** | 0.98*** | 1.00*** | 0.96*** | -0.58NS | 0.94*** | 0.58NS | 1.00 |
